# Use of beta-lactams antibiotics in ICU patients

**DOI:** 10.1186/2197-425X-3-S1-A399

**Published:** 2015-10-01

**Authors:** M-P Damas, C Vercheval, M Nys, B Lambermont, A Ancion, P Damas

**Affiliations:** CHU Liège, Liège, Belgium

## Introduction

Large spectrum antibiotics have to be considered as first treatment of infection in ICU patients.

## Objectives

To evaluate the antibiotic treatments in ICU patients and to compare treatments on admission and during ICU stay.

## Methods

A Six-month prospective study in 5 ICUs of a hospital totalizing 44 ICU beds. Antibiotic treatments were divided in three groups: treatment started before ICU admission (1), treatments started the day of ICU admission (2) and treatments initiated during ICU stay (3). The type of beta-lactam antibiotics (BA) used was listed at the start of treatment and after 3 days.

## Results

459 treatments given to 363 patients among 1131 hospitalized patients were analyzed. There were respectively 131, 167 and 161 treatments in group 1, 2 and 3. After 3 days, 79 (17.2%) treatments could be stopped because of no infections and 129 (28.1%) were changed mostly for streamlining. Table 1 shows the number of treatment with BA according to groups and to the time of treatment.

**Table 1:** Table 1

Type of BA	Before admission (group 1)	On admission (group 2)	During ICU stay (group 3)	After 3 days n = 96 (group 1)	After 3 days n = 133 (group 2)	After 3days n = 148 (group 3)
Ampicilline- Amoxicilline - Temocilline	2 (1,5%)	2 (1,2%)	3 (1,9%)	4 (4,2%)	9 (6,8%)	10 (6,8%)
Penicillin	1 (0,8%)	1 (0,6%)	0 (0%)	1 (1%)	4 (3%)	0 (0%)
Flucloxacillin	8 (6%)	0 (0%)	10 (6%)	11 (11,5%)	5 (3,8%)	11 (6,8%)
Amoxi-Clav	15 (11,5%)	35 (21%)	15 (9,3%)	1 (1%)	13 (9,8%)	7 (4,7%)
Pipera-Tazo	42 (32,1%)	54 (32,3%)	32 (19,9%)	17 (17,7%)	21 (15,8%)	13 (8,8%)
Cephal 2G	3 (2,3%)	5 (3%)	9 (5,6%)	8 (8,3%)	11 (8,3%)	15 (10,1%)
Cephal 3G	14 (10,7%)	34 (20,4%)	35 (21,7%)	19 (19,8%)	20 (15%)	26 (17,6%)
Cephal 4G	7 (5,3%)	1 (0,6%)	2 (1,2%)	2 (2,1%)	1 (0,8%)	1 (0,7%)
Carbapenem	17 (13%)	15 (8,9%)	19 (11,8%)	10 (10,4%)	12 (9%)	14 (9,5%)

There was a tendency to use less BA during ICU stay, the difference was significant only at the start of treatment (p = 0.027). If we consider ampicillin, temocillin, penicillin, flucloxacillin and 2nd -generation cephalosporin as molecule with narrow spectrum of activity, these molecules were significantly more used after three days of treatment (p < 0.0001). Interestingly this difference already existed at the start of treatment when group 3 was compared to the two others (p = 0.0159). Among the 459 treatments, there were, in 49 patients, 51 inappropriate treatments according to the bacteriological results, 16 (12%) in group 1, 17 (10%) in group 2 and 18 (11%) in group 3 (p = 0.857). The ICU mortality was not different between those patients (11/49 = 22%) and the others (77/314 = 24.5%) (p = 0859).

## Conclusions

Beta-lactam antibiotics remain the cornerstone of antibacterial therapy. A cautious strategy with narrow spectrum beta-lactam antibiotics could be supported in ICU patients when possible.

**Table 2:** Table 2

TOTAL	109 (83,2%)	147 (88%)	125 (77,6%)	73 (76%)	96 (72,2%)	97 (65,5%)

